# Common integration sites of published datasets identified using a graph-based framework

**DOI:** 10.1016/j.csbj.2015.11.004

**Published:** 2015-11-29

**Authors:** Alessandro Vasciaveo, Ivana Velevska, Gianfranco Politano, Alessandro Savino, Manfred Schmidt, Raffaele Fronza

**Affiliations:** a Department of Translational Oncology, National Center for Tumor Diseases and German Cancer Research Center, Im Neuenheimer Feld 581, 69120 Heidelberg, Germany; b Department of Control and Computer Engineering, Politecnico di Torino, Corso Duca degli Abruzzi 24, 10129 Torino, Italy

## Abstract

With next-generation sequencing, the genomic data available for the characterization of integration sites (IS) has dramatically increased. At present, in a single experiment, several thousand viral integration genome targets can be investigated to define genomic hot spots. In a previous article, we renovated a formal CIS analysis based on a rigid fixed window demarcation into a more stretchy definition grounded on graphs. Here, we present a selection of supporting data related to the graph-based framework (GBF) from our previous article, in which a collection of common integration sites (CIS) was identified on six published datasets. In this work, we will focus on two datasets, IS_RTCGD_ and IS_HIV_, which have been previously discussed. Moreover, we show in more detail the workflow design that originates the datasets.

Specifications Table

**Table:** 

Subject area	Computational biology, systems biology
More specific subject area	Gene therapy, integrational mutagenesis analysis
Type of data	Table, image, dataset
How data was acquired	In silico experiments
Data format	Analyzed datasets, analyzed Excel tables, PNG files
Experimental factors	Integration sites datasets were analyzed with a new computational method for common integration sites identification
Experimental features	A proposed set of common integration sites from two published integration sites datasets (see [1])
A pathway enrichment analysis is also reported	
Data source location	Heidelberg, Germany
Data accessibility	Data is with this article and in ref. [1]

Value of the data

• The analyzed dataset here provided can be used as benchmark to compare the results of the graph modeling approach for CIS identification and analysis implemented in software tools.
• Graph modeling approach to the identification of common integration sites.
• Validation of the graph-based framework (GBF) against well-known datasets.
• Detailed illustrated procedure for the identification of CIS via GBF.

## 1 Data

**Fig. 1:**
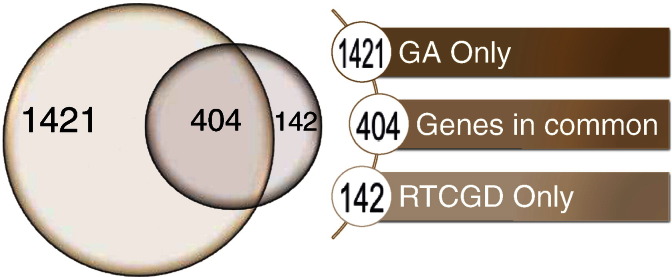
Venn diagram of the gene atmosphere of all identified CIS from the RTCGD dataset using the GBF (graph-based framework) [1] and using the SWM (standard window method) [2].

The dataset containing the identified CIS from the Retroviral Tagged Cancer Gene Database (RTCGD) [6] is provided in Table 1 Appendix A and it is obtained by using a Cytoscape 2.8 plugin, which implements some of the features of the GBF method (see how to retrieve the code in [1]). The other datasets are collected using a normal Internet browser. Fig. 1 shows a Venn diagram in which two datasets are compared. The first dataset is the collection of all the genes found with the GBF method, while the second dataset is the list of genes provided by RTCGD which uses the standard window method (SWM) to identify CIS and the next gene approach (NGA) to discover and associate an annotated gene to the identified CIS. For further details about the two approaches, see [1]. With the GBF method, it is possible to discover 1421 genes which are not present in the RTCGD dataset. Only 142 genes were not discovered by the GBF method while they are present in the RTCGD gene list, and 404 of the genes can be found by both methods.

## 2 Experimental design, materials and methods

### 2.1 Experiment workflow

**Fig. 2:**
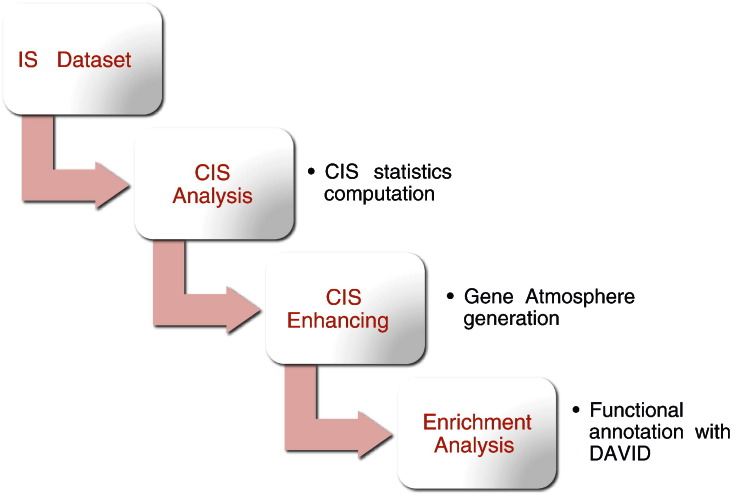
Workflow of the full analysis process: starting from the raw dataset to the functional analysis.

The workflow of the analysis is depicted in Fig. 2. The input is a dataset composed of a list of integration sites (IS). The graph-based framework (GBF) presented in [1] is adopted to perform all the following analyses. The first step is the CIS identification and the computation of some statistics for every CIS. Further steps are optional but they have to follow the order. The second step consists of enhancing the CIS dataset with information from genomic annotated data. This step generates the gene atmosphere (GA) dataset as shown in Table 2 Appendix A. Using the GA dataset, the next step consists of the functional analysis, as shown in Table 3 Appendix A.

### 2.2 Data preparation

The dataset used for the analysis should contain few attributes in order to be properly analyzed by the GBF method. Some of these attributes are mandatory and they are shown in Table 1. The mandatory attributes for the CIS enhancing phase are shown in Table 2.

### 2.3 Common integration sites identification

**Fig. 3:**
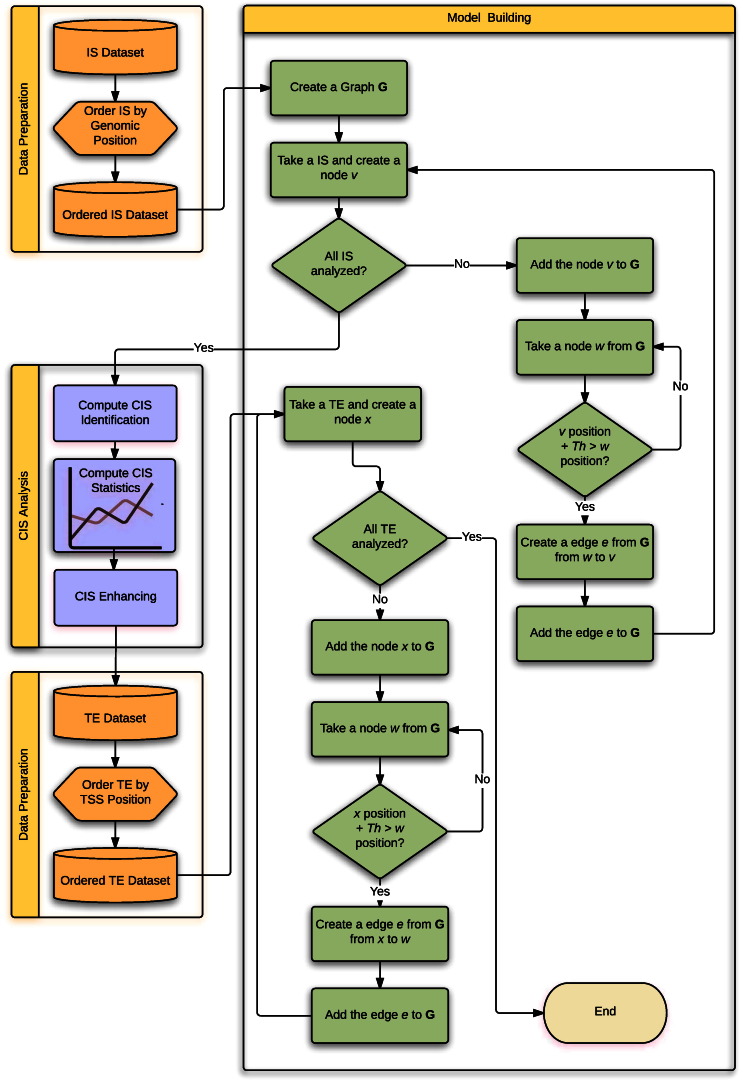
Flowchart of the main method for the identification and enhancing of CIS using the graph-based framework.

The method presented in [1] allows the identification of CIS on the basis of very few attributes found in the dataset under analysis (see Table 1). Fig. 3 shows the flowchart of the global method that builds the model and identifies the CIS with their statistics.

Starting from the dataset containing the integration sites (IS dataset), it is convenient to order the dataset according to the integration position to improve the algorithm efficiency. This is the data preparation part (Table 1). Afterwards, as depicted in Fig. 3, the building of the model starts creating an empty graph. For every IS present in the dataset, a node is created and added to the graph. A nested loop checks if all the vertices instantiated in the graph are at a distance below a certain threshold from the current IS previously added as a node to the graph itself. An edge connecting two nodes of the same type (i.e. two IS nodes) is created and added to the graph if the distance is lower than the threshold. When all the IS from the dataset are analyzed, the main loop terminates and the graph is ready to be analyzed by the main algorithm for CIS identification. This algorithm can be implemented in different ways (e.g. an algorithm that extracts the connected components (CC) from an undirected and disconnected graph). An efficient version of this algorithm is presented in [3].

### 2.4 Common integration sites statistics computation

When the CIS identification is performed, a set of statistics are computed. The most interesting statistics are presented in Table 3. For further details about how the statistics have been computed, see Paragraph 2.6 in [1].

### 2.5 Common integration sites enhancing

Optionally, an enhancing of the CIS dataset can follow. The purpose is to link each IS with its neighborhood on the genome retrieving annotations present in online databases. Here, we used a normal Internet browser to perform queries accessing annotated data provided online by the BioMart database [4]. The dataset resulting from this step is shown in Table 2 Appendix A, which provides a list of transcriptional elements (TE) composing the GA of all CIS identified with the previous step. As shown in the flowchart in Fig. 3, the process that builds the GA is similar to the process that build the IS graph. The IS nodes in the graph are linked with the TE nodes if the distance on the genome is below a certain threshold.

### 2.6 Functional annotation using a GA list

If the previous step is performed, a functional annotation using DAVID [5] may follow. This is the last step of the main workflow shown in Fig. 2. Here, we perform this step using the RTCGD dataset and the output is shown in Table 3.

### 2.7 CIS properties computed in the Cytoscape prototype

CIS number

Integer value given to a CIS by the plugin.

CIS name

Name of the CIS as it appears in the tabular exported file. It is a composition of the chromosome and the CIS number.

CIS order

Number of IS that compose the CIS.

CIS average position

Approximate CIS position *p_A_* calculated as $p_{A}=\frac{{\mathrm{IS}}_{\mathrm{first}}+\;{\mathrm{IS}}_{\mathrm{last}}}{2}$; IS_first_ and IS_last_ are the positions on the chromosome of the first and last IS in the CIS.

CIS median position

Approximate CIS position *p_M_* calculated sorting the *n* IS as they appear on the chromosome:

(1) $p_{M}=\frac{IS_{\left(n+1\right)}}{2}$ if *n* is odd or
(2) $p_{M}=\frac{IS_{\left(\frac{n}{2}\right)}+IS_{\left(\frac{n}{2}+1\right)}}{2}$ if *n* is even.

IS_(*i*)_ is the position of the *i*th IS of the CIS. For CIS with an asymmetric distribution of the IS, this approximation gives a more precise estimation.

CIS entropy

If the number of different labels (entropy label) found in the CIS is *n* and the order is *O*, the entropy value is computed as

$$E_{CIS}=\sum_{i=1}^{n}\frac{\frac{n_{i}}{O}\log\frac{n_{i}}{O}}{\log n}$$

where *n*_*i*_ is the number of IS labelled with *i*.

Normalized entropy

If the number of different labels (entropy label) found in the entire dataset is *N* and the order of the CIS is *O*, the entropy value is computed as

$${NE}_{CIS}=\sum_{i=1}^{N}\frac{\frac{n_{i}}{O}\log\frac{n_{i}}{O}}{\log N}$$

where *n*_*i*_ is the number of IS labelled with the label *i*.

CIS *p* value

See the subsection “Statistical model, *p*-value and log-likelihood ratio test” in [1]

CIS loglike ratio

See the subsection “Statistical model, *p*-value and log-likelihood ratio test” in [1]

## Figures and Tables

**Table 1:** Mandatory attributes of the input dataset for the identification of CIS using the GBF method.

Attributes	Description
Chromosome number	The ordinal number of the chromosome in which the integration event was found
Insertion site position	The position on the genome: a very long integer number representing the base pair where the virus was integrated
Entropy label (e.g. Kind of tumor, virus type)	Meta-information used for the computation of the CIS entropy. It is a label that represents a factor of the experiment. For example, it could be the tumor model or type from which the IS has been associated

**Table 2:** Mandatory attributes of the input dataset for enhancing analysis using annotated genomic data against the GBF method.

Attributes	Description
Chromosome number	The ordinal number of the chromosome in which the TSS of the gene is located
Transcription start site	The position on the genome: a very long integer number representing the base pair where transcription starts at the 5′-end of a gene sequence

**Table 3:** Computed statistics for CIS.

Statistic	Description
CIS order	The total number of IS present in the CIS
CIS dimension	The number of base pairs that contain all the IS belonging to a single CIS (see Section 2.7 for details)
CIS *p*-value	The *p*-value associated to the CIS. See Paragraph 3.6 in [1] for a comprehensive explanation
CIS entropy	The entropy of the CIS based on the label from the input dataset (e.g. tumor type, virus type). See paragraph 3.6 in [1] and Section 2.7

## Appendix A Supplementary data

Table 1 — Identified CIS and their statistics using the GBF method on the RTCGD dataset (only CIS of order ≥ 4). Table 2 — Gene atmosphere of the identified CIS using the GBF method on the RTCGD dataset. For each gene the transcription start site (TSS) is reported. Table 3 — Functional analysis using DAVID. Two gene lists analyzed: the genes found both with GBF and SWM on the RTCGD dataset, and the genes found only with GBF. Table 4 — CIS found on HIV dataset (see [1]) only CIS of order ≥ 10.
